# Quantitative Analysis of Sulfur Elements in Mars-like Rocks Based on Multimodal Data

**DOI:** 10.3390/s25144388

**Published:** 2025-07-14

**Authors:** Yuhang Dong, Zhengfeng Shi, Junsheng Yao, Li Zhang, Yongkang Chen, Junyan Jia

**Affiliations:** 1 School of Mechanical, Electrical & Information Engineering, Shangdong University, Weihai 264209, China; yuhangdong@mail.sdu.edu.cn (Y.D.); zhengfengshi@mail.sdu.edu.cn (Z.S.); jsyao@mail.sdu.edu.cn (J.Y.); yongkangchen@mail.sdu.edu.cn (Y.C.); junyanjia@mail.sdu.edu.cn (J.J.); 2 Shangdong Key Laboratory of Intelligent Electronic Packaging Test and Application, Shangdong University, Weihai 264209, China

**Keywords:** Mars, LIBS, multimodal learning, sulfur, feature selection, quantitative analysis

## Abstract

The Zhurong rover of the Tianwen-1 mission has detected sulfates in its landing area. The analysis of these sulfates provides scientific evidence for exploring past hydration conditions and atmospheric evolution on Mars. As a non-contact technique with long-range detection capability, Laser-Induced Breakdown Spectroscopy (LIBS) is widely used for elemental identification on Mars. However, quantitative analysis of anionic elements using LIBS remains challenging due to the weak characteristic spectral lines of evaporite salt elements, such as sulfur, in LIBS spectra, which provide limited quantitative information. This study proposes a quantitative analysis method for sulfur in sulfate-containing Martian analogs by leveraging spectral line correlations, full-spectrum information, and prior knowledge, aiming to address the challenges of sulfur identification and quantification in Martian exploration. To enhance the accuracy of sulfur quantification, two analytical models for high and low sulfur concentrations were developed. Samples were classified using infrared spectroscopy based on sulfur content levels. Subsequently, multimodal deep learning models were developed for quantitative analysis by integrating LIBS and infrared spectra, based on varying concentrations. Compared to traditional unimodal models, the multimodal method simultaneously utilizes elemental chemical information from LIBS spectra and molecular structural and vibrational characteristics from infrared spectroscopy. Considering that sulfur exhibits distinct absorption bands in infrared spectra but demonstrates weak characteristic lines in LIBS spectra due to its low ionization energy, the combination of both spectral techniques enables the model to capture complementary sample features, thereby effectively improving prediction accuracy and robustness. To validate the advantages of the multimodal approach, comparative analyses were conducted against unimodal methods. Furthermore, to optimize model performance, different feature selection algorithms were evaluated. Ultimately, an XGBoost-based feature selection method incorporating prior knowledge was employed to identify optimal LIBS spectral features, and the selected feature subsets were utilized in multimodal modeling to enhance stability. Experimental results demonstrate that, compared to the BPNN, SVR, and Inception unimodal methods, the proposed multimodal approach achieves at least a 92.36% reduction in RMSE and a 46.3% improvement in R^2^.

## 1. Introduction

Previous studies have revealed the widespread distribution of various sulfate minerals on the Martian surface [1,2], which provide critical scientific evidence for investigating Martian habitability, paleo-hydrological conditions, and atmospheric evolution [3,4].

In July 2020, China successfully launched the Tianwen-1 Mars probe [5]. In 2021, the Zhurong rover of the Tianwen-1 mission reached the Martian surface and commenced its exploration operations [6]. Around 2028, China plans to launch the Tianwen-3 probe and achieve the Mars sample return to Earth. In order to complete the detection tasks, Zhurong is equipped with 13 scientific payloads, including a multispectral camera (Xi’an Institute of Optics and Precision Mechanics, Chinese Academy of Sciences, Xi’an, China) and the Mars Surface Composition Detector (MarSCoDe, Shanghai Institute of Technical Physics, Chinese Academy of Sciences, Shanghai, China). These instruments are designed to acquire high-resolution images of the Martian surface, subsurface geological features, and climatic data, thereby providing critical datasets for in-depth investigations of Mars. Particularly noteworthy is the LIBS instrument within MarSCoDe, which plays a pivotal role in determining the chemical composition of Martian surface materials [7].

LIBS is an atomic emission spectroscopy technique that generates localized plasma by irradiating a sample surface with a high-energy pulsed laser beam [8]. Since nearly all elements emit characteristic spectral lines when excited to a plasma state, LIBS enables both qualitative and quantitative analyses of elemental compositions in Martian surface materials through detailed spectral interpretation. This technique demonstrates distinct advantages over other chemometric methods. First, it imposes minimal sample pretreatment requirements, accommodating solid, liquid, or gaseous states. This eliminates the need for additional chemical processing when analyzing Martian surface materials, thereby simplifying analytical procedures. Second, as a non-contact detection method, LIBS offers remote detection capabilities, particularly suited for planetary exploration missions. With high sensitivity and resolution, it enables the accurate identification of trace elements and compounds in Martian surface materials [9].

The Zhurong rover landed in southern Utopia Planitia, where partial sulfates were detected in its exploration area, confirming previous remote sensing predictions. In LIBS quantitative analysis, calibration-free models and calibration-based models represent two commonly employed analytical approaches [10].

Calibration-free models require no prior knowledge of sample concentrations. These models predict sample composition through physicochemical principles (e.g., plasma physics and elemental spectral behavior) [11]. Calibration-based models rely on samples with known concentrations to establish calibration curves, typically categorized into univariate and multivariate models [12]. Univariate models utilize the relationship between the intensity of a single spectral line and sample concentration, generally assuming a linear correlation between spectral intensity and concentration. Multivariate models consider multiple spectral line intensities and their relationships with concentrations, typically requiring complex mathematical methods to process multidimensional data, but generally demonstrating superior accuracy and applicability compared to univariate models.

Yang et al. successfully implemented sample classification analysis for multi-detection-distance LIBS spectra by constructing a Convolutional Neural Network (CNN) model. Their study demonstrated that CNN models achieve superior overall classification accuracy compared to traditional machine learning methods, particularly when trained with large sample datasets [13]. Wang et al.’s team innovatively employed a Deep Adaptive Deep Neural Network (DADNN) with a cross-device transfer learning strategy to transfer spectral prior knowledge from the ChemCam Mars instrument to the SDU-LIBS ground system, effectively addressing cross-platform data distribution discrepancies [14]. Weritz et al. established a calibration model between sulfur characteristic line intensity and concentration using LIBS technology, achieving quantitative detection of sulfur content in concrete [15]. Fang et al. developed an ensemble learning model (HEL) for full-spectrum LIBS quantitative regression analysis through a Bayesian weighting strategy that integrates the advantages of four heterogeneous sub-models, attaining enhanced accuracy and stability [16]. Duponchel, Ludovic’s team investigated whether inverse models should be prioritized in LIBS quantitative analysis, experimentally demonstrating that inverse models generally provide more accurate predictions [17]. A heterogeneous stacked ensemble learning model comprising a two-stage architecture was proposed by Zeng et al.’s team, achieving improved performance in the quantitative analysis of complex chemical samples [18].

However, most LIBS quantitative analyses primarily focus on cationic elements. Gu et al. achieved quantitative analysis of Mn in complex alloy matrices by extracting spatiotemporal features from LIBS spectra through multi-perspective feature extraction and backward differentiation methods [19]. Naser and Hamad’s team estimated heavy metal concentrations (Cr, Cd, Zn, Pb, and Cu) in gallstone samples collected from various hospitals using calibration curves constructed between LIBS emission line intensities and AAS-measured elemental concentrations, achieving relative errors within 1.5–7.8% [20]. Sui et al. integrated ultrasonic nebulization-assisted equipment with conventional LIBS systems, realizing high-sensitivity, stable, and rapid online detection of metallic elements in seawater [21].

Quantitative analysis of anionic elements, such as sulfur, remains challenging due to weak spectral line intensities in LIBS spectra. Traditional quantitative models typically require extensive spectral datasets for effective training, with performance significantly deteriorating under small-sample conditions [22,23]. To address these challenges, this study proposes a multimodal approach for the quantitative analysis of sulfur in Martian rocks through the synergistic utilization of spectral features. Specifically, the method first employs a modified Vision Transformer (ViT) network to process infrared spectral data for binary sample classification (high vs. low sulfur content). Subsequent quantitative analysis utilizes a custom-built Multimodal Convolutional Neural Network (MCNN) that strategically integrates atomic emission features from LIBS spectra with molecular vibrational information from infrared spectroscopy while implementing distinct concentration-dependent modeling for each predefined group. Notably, the high-dimensional nature of LIBS spectra introduces overfitting risks in regression tasks. To mitigate this issue, a feature selection scheme is implemented on LIBS spectral data prior to multimodal fusion, with selected feature subsets subsequently fed into the multimodal model [24,25,26]. Finally, comprehensive validation was conducted to verify the effectiveness of the proposed method.

## 2. Materials

**Figure 1:**
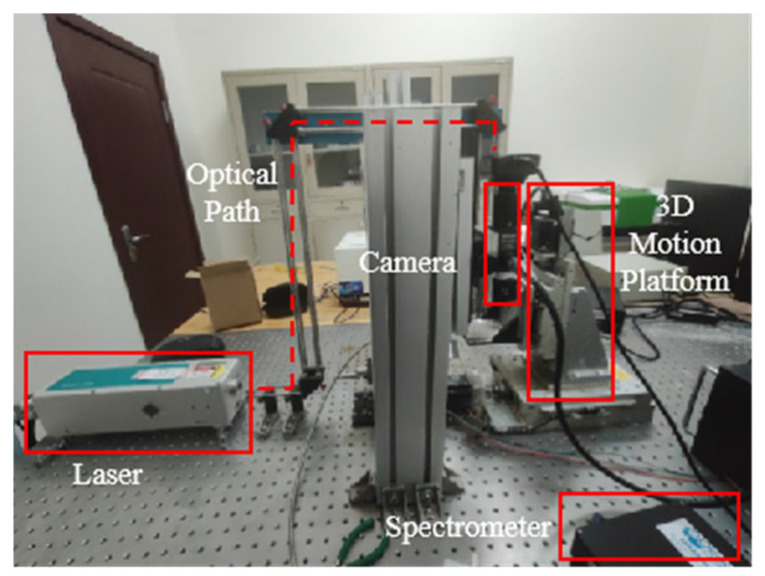
Physical drawing of LIBS system.

The spectral instruments employed in this study are laboratory-replicated systems, consisting primarily of a LIBS spectral acquisition platform and an infrared spectroscopy platform. As illustrated in Figure 1, the LIBS platform consists of a laser source, optical path, three-axis motion stage, spectrometer, imaging camera, and computer. The laser system used in this study is a Q-switched Nd:YAG laser (DAWA200, Beamtech Optronics, Beijing, China). The DAWA200 laser supports both external and internal triggering modes; in this experiment, it was synchronized with the spectrometer under external triggering conditions at the 1064 nm wavelength, with a single-pulse energy of up to 200 mJ, a repetition rate of 1 Hz, and a pulse duration of less than 8 ns. After reflection, the laser beam passes through a dichroic mirror and is then focused by a 200 mm focal length, 10× magnification microscope objective, which is connected to a microscope camera, preceded by a 0.5× tube lens. Plasma radiation is collected by two plano-convex lenses, each with a focal length of 50 mm. The collected radiation is then transmitted through a Y-shaped optical fiber. This fiber guides the radiation to the spectrometer. The spectrometer used is a model produced by Avantes (Apeldoom, The Netherlands), equipped with a CCD detector (AvaSpec-ULS2048, Avantes BV, Apeldoom, The Netherlands). The spectrometer covers a spectral range of 200–860 nm, with a resolution of 0.3 nm, a minimum integration time of 1.05 ms, and a delay time of 1 µs. To enable multi-point spectral acquisition on the same sample, a three-axis motion stage was employed. This system incorporated dual five-phase motor drives: the KR-55ME-2Z from KOHZU Inc. (Tokyo, Japan) and the PMDO7CV from VEXTA/Oriental Motor Co., Ltd. (Tokyo, Japan). After calibration and optimization, the positioning accuracy of the motion stage along the X-, Y-, and Z-axes was improved to <100 nm. Additionally, a Nikon camera module and an illumination system, sharing the laser optical path, were integrated for real-time surface imaging. This configuration ensured precise laser targeting and continuous focus verification during stage movements.

**Figure 2:**
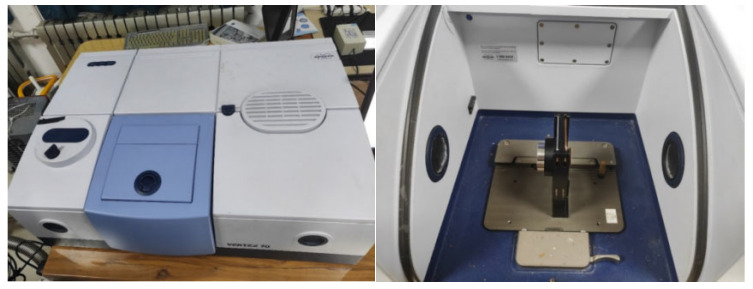
Infrared spectrometer and sample stage.

The infrared spectral acquisition platform utilized a Fourier-transform infrared (FTIR) spectrometer (Vertex70 model, Bruker Corporation, Billerica, MA, USA), operating in the spectral range of 400–4000 cm^−1^ with a resolution of 4 cm^−1^. Sample and background spectra were acquired by averaging 32 co-added scans of a pure aluminum mirror coated with a SiO_2_ protective film, with the sample placed inside the spectrometer. Post-processing, including atmospheric compensation and baseline correction, was performed using OPUS 6.0 software, followed by spectral data recording. The configuration of the FTIR spectrometer and sample stage is illustrated in Figure 2.

This study primarily focuses on sulfur, establishing a dataset of evaporite-matrix binary mixtures for combined analysis using LIBS and infrared spectroscopy. The dataset comprises 28 synthetic samples designed for quantitative sulfur analysis. These samples were prepared by proportionally mixing simulated Martian rock matrices with various evaporite minerals to systematically investigate the correlation between elemental content and spectral characteristics. Material selection carefully considered three critical factors: rock matrix composition, mineral hydration states, and cationic element interference. Two igneous rocks (basalt and andesite) were employed as geological matrices, combined with six evaporite minerals, including anhydrous calcium sulfate (CaSO_4_), gypsum (CaSO_4_·2H_2_O), plaster of Paris (CaSO_4_·0.5H_2_O), potassium sulfate (K_2_SO_4_), magnesium sulfate (MgSO_4_), and ferrous sulfate (FeSO_4_). The specific sample configurations are systematically categorized in Table 1.

**Figure 3:**
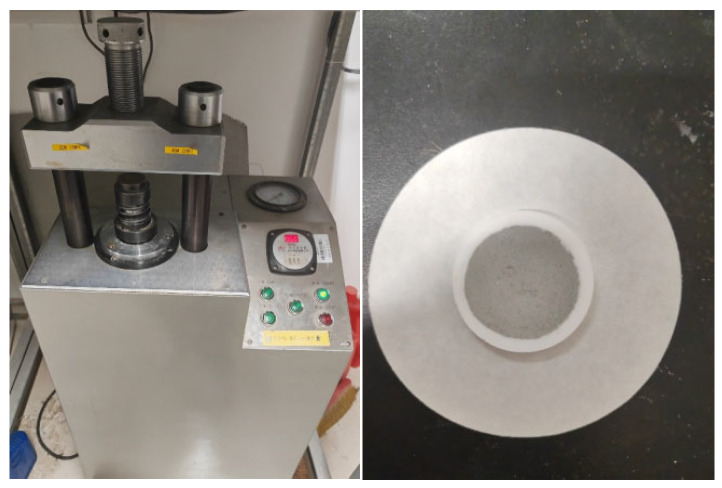
Tablet press and pressed sample physical drawing used in LIBS experiment.

Each rock matrix was prepared using Chinese national standard reference materials (certified reference materials, CRMs) accompanied by authentication certificates. These certificates provide detailed specifications, including elemental composition, abundance data, analytical methodologies, and measurement uncertainties, with selected specifications summarized in Table 2. It should be noted that, due to the large size of the complete specification table, it is not appropriate to list all of its contents in the main text. Table 2 presents only a subset of the specifications, with the complete specification table provided in Supplementary Materials (Table S1). All samples were processed into powdered form (Figure 3) prior to mounting on the LIBS sample stage. Analytical sequences were initiated by triggering laser pulses under controlled ambient conditions. For statistical reliability, five distinct spots were ablated per sample, with three spectral acquisitions averaged at each spot to generate representative spectral data.

For infrared spectroscopic analysis, homogenized sample powders were thoroughly mixed with potassium bromide (KBr) at an optimal mass ratio and pulverized using an agate mortar to ensure particle uniformity. Following homogenization, the mixtures were compressed into transparent pellets (13 mm diameter) to enable infrared transmission measurements. The pellets were subsequently analyzed under standardized instrumental conditions, yielding a dataset dimensionally consistent with the corresponding LIBS dataset.

## 3. Methods

**Figure 4:**
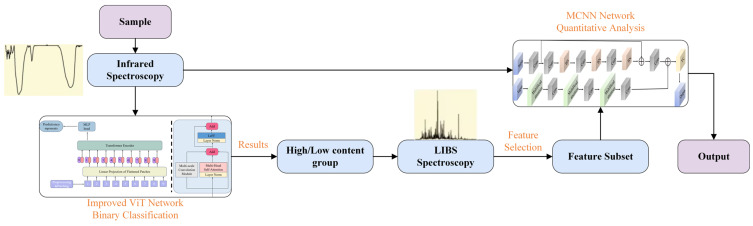
Research workflow framework.

The primary methodology employed in this study can be summarized as classification followed by regression. As illustrated in Figure 4, samples are first categorized into low-concentration and high-concentration groups based on infrared spectral data and their sulfur content. Subsequently, dual-input regression models are established for each group, where CNNs with residual structures extract local emission peak features from LIBS spectra, while the infrared channel employs multi-head self-attention mechanisms combined with CNNs to capture broad spectral band correlations. Finally, a fully connected layer integrates decisions from both spectral modalities to predict sulfur content.

For the classification task, this study utilizes a modified ViT network, while the quantitative analysis employs a custom-built MCNN architecture. The presentation proceeds systematically: first introducing the modified ViT model for classification, followed by the MCNN architecture for quantitative analysis, and concluding with the feature selection methodology.

### 3.1. ViT Architecture and Modifications

**Figure 5:**
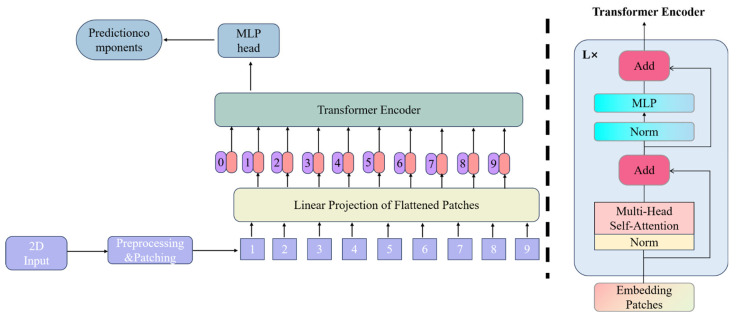
ViT network architecture diagram.

The Vision Transformer (ViT) [27], a variant of the Transformer model designed for image processing, is schematically illustrated in Figure 5. This model primarily utilizes the encoder component of the Transformer for data processing. Unlike traditional convolutional neural networks, the core methodology of ViT involves first converting images into a series of vector sequences, then extracting features from these sequences using the Transformer encoder, and finally predicting classification or regression outcomes using multilayer perceptrons (MLPs). The workflow consists of four primary stages:

(1) Patch embedding: Input images are divided into fixed-size patches (typically 16 × 16 pixels), which are then projected into 1D vector sequences via linear projection layers.

(2) Positional encoding: To account for lost spatial information during vectorization, positional encoding matrices (with dimension N × D, where N represents the sequence length) are additively combined with patch embeddings. Crucially, this additive operation preserves the original vector dimensions.

(3) Layer normalization (LN) and multi-head self-attention: The encoded sequence undergoes feature extraction through stacked Transformer encoders. Layer normalization stabilizes training by mitigating gradient vanishing/explosion while improving convergence speed and generalization capacity. The multi-head self-attention mechanism enables multi-perspective feature learning, with parallel attention heads capturing distinct feature subspace representations. This architectural design enhances feature richness and model robustness against single-head failures. Both LN and attention operations maintain dimensional consistency.

(4) Multilayer perceptron (MLP): The MLP module performs dimensional scaling through expansion–reduction transformations, ensuring identical input–output dimensions for encoder stacking. The final MLP layer functions similarly to fully connected layers in CNNs, generating final classification or regression predictions.

The ViT effectively models long-range dependencies in images through its Transformer architecture and extracts global features via multi-head self-attention mechanisms, demonstrating superior expressive power and flexibility over traditional CNNs. Additionally, regularization strategies, such as layer normalization, stabilize the training process and enhance model generalization. Consequently, a ViT-based framework is employed for the classification analysis of infrared spectral data in this study. However, sulfur-specific absorption peaks in infrared spectra manifest as localized narrowband signals [28], while the global attention mechanism of the native ViT’s exhibits insufficient sensitivity to local features. Moreover, the MLP introduces computational redundancy during the processing of high-dimensional spectral sequences. To address these limitations, targeted modifications to the encoder component of the ViT architecture are proposed.

To preserve the global modeling capabilities of attention mechanisms while enhancing inductive biases for local pattern recognition, multi-scale convolutional modules are integrated with the ViT framework in this work. Convolutional operations, characterized by localized receptive fields and weight-sharing mechanisms, effectively extract structural local features, thus compensating for the shortcomings of the ViT in local detail modeling [29]. Furthermore, the translation equivariance inherent in CNNs enables robust adaptation to spatially varying features, thereby strengthening model generalization. The efficient local computation paradigm of convolutional layers also improves inference speed, enhancing overall model efficiency and performance. By synergistically combining the localized feature extraction of CNNS with the global representation learning of ViT, our hybrid architecture achieves an optimized balance between computational efficiency, generalization capacity, and predictive performance.

**Figure 6:**
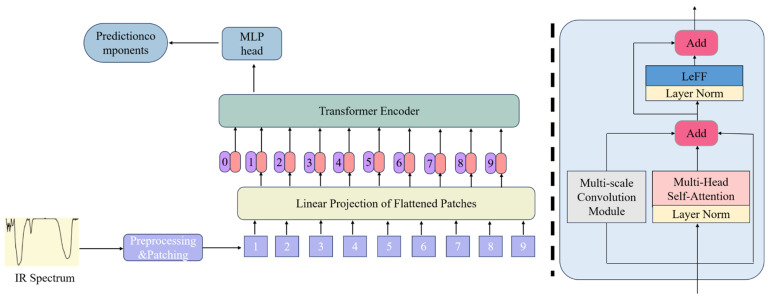
Improved ViT architecture diagram.

**Figure 7:**
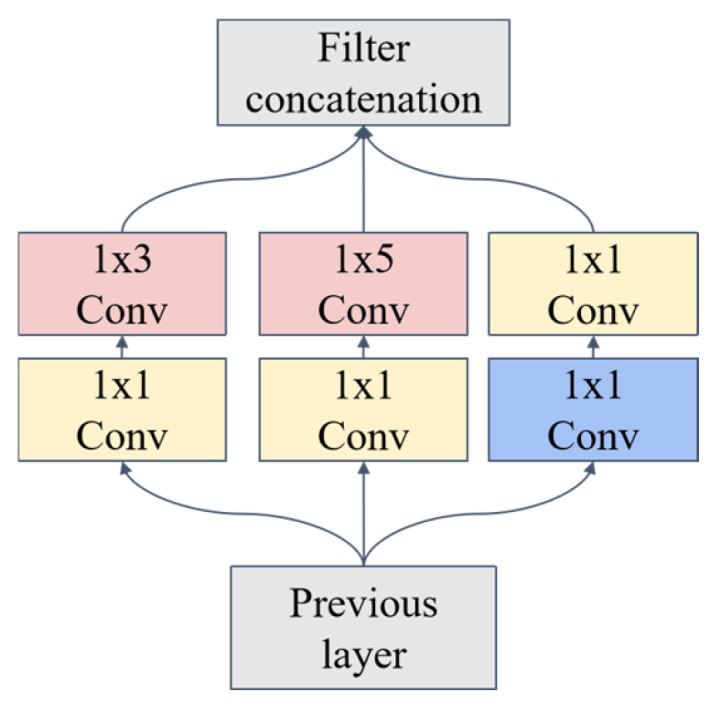
Multi-scale convolutional module.

An auxiliary branch is introduced into the multi-head self-attention component of the ViT encoder in this study, as illustrated in Figure 6. Inspired by the Inception architecture [30], this branch integrates convolutional layers (Conv), batch normalization (BN), max pooling, and LeakyReLU activation functions, as shown in Figure 7. Multi-scale convolutional kernels operate in parallel to extract local spectral features across varying receptive fields, capturing fine peak shapes from adjacent wavenumbers and the transitional characteristics between absorption peaks and background signals simultaneously. The inherent translation invariance of convolutional operations mitigates interference from wavenumber shifts caused by spectral baseline drift, while the introduced locality inductive bias helps reduce overfitting risks under small-sample conditions. During encoder processing, both attention computations and convolutional operations are executed concurrently to enhance the local inductive bias of the network. Within this branch, convolutional layers extract localized features, while batch normalization accelerates convergence. The LeakyReLU activation function addresses neuron mortality by enabling gradient propagation for negative inputs while introducing nonlinearity to improve fitting capacity.

**Figure 8:**
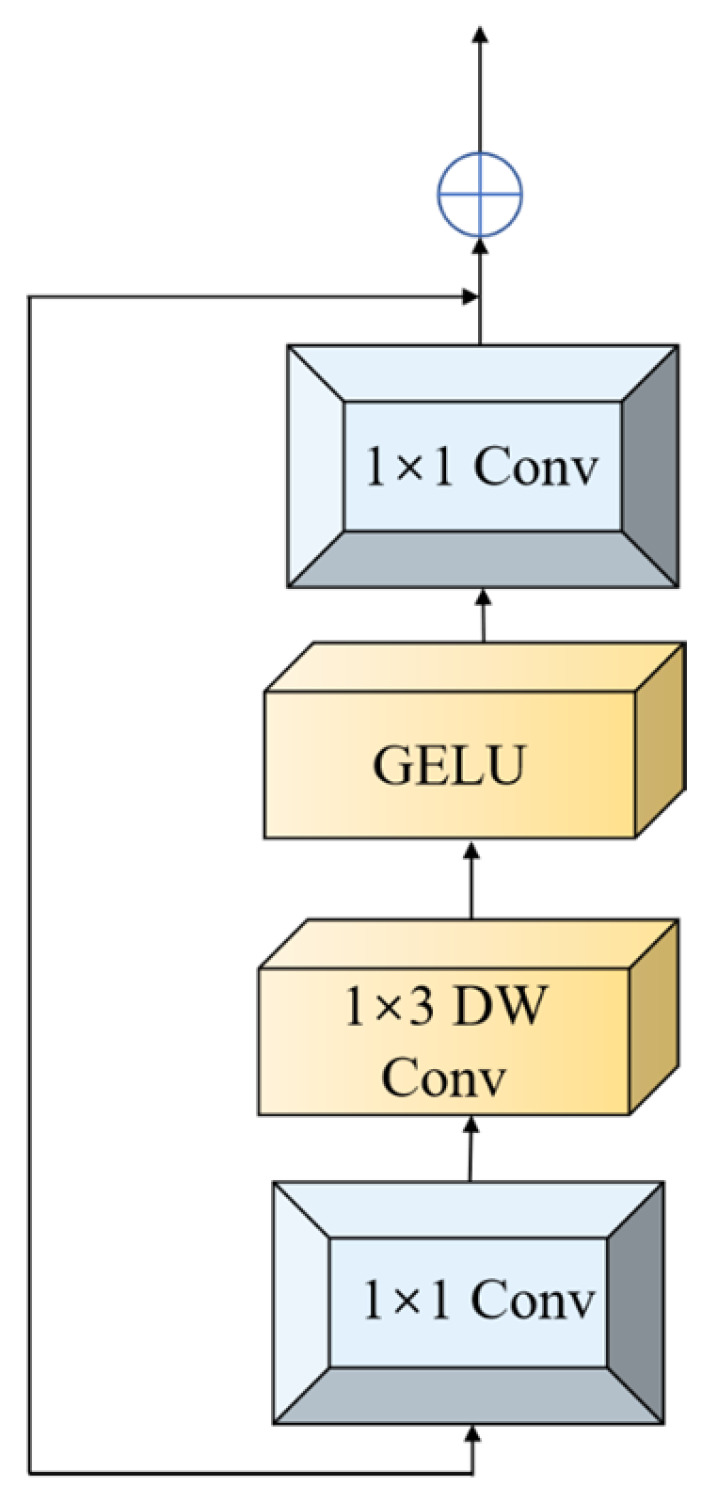
Leff module.

Beyond the auxiliary branch, modifications to the ViT’s MLP component were implemented (Figure 8). Drawing insights from CeiT [31] and LocalViT [32], feature extraction is enhanced through a stacked structure comprising 1 × 1 convolutions, 1 × 3 depth-wise (DW) convolutions, and additional 1 × 1 convolutions. Residual connections are incorporated to prevent network performance degradation. The embedded depth-wise separable convolutions strengthen local wavenumber continuity modeling and dynamically prioritize critical sulfur-related feature channels. Skip connections enable cross-level integration of shallow convolutional details and deep attentional features, reducing computational complexity and improving discriminative capacity for subtle concentration variations simultaneously.

### 3.2. MCNN Architecture

**Figure 9:**
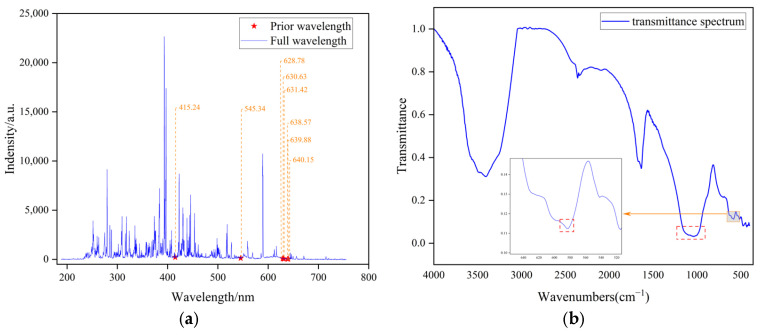
Prior knowledge of the spectrum. (**a**) The characteristic spectral lines of sulfur in LIBS spectra. (**b**) The characteristic spectral lines of sulfur in infrared spectra.

Spectral data, as a specialized data modality, inherently encapsulate rich material-specific information and structural characteristics across various forms, including LIBS, IR spectroscopy, and Raman spectroscopy. However, the detection capabilities of spectroscopic techniques are inherently constrained by their physical interaction mechanisms, which result in selective sensitivity to specific chemical compositions and spatial configurations. For nonmetallic elements, such as sulfur, limitations arise from low ionization efficiency during plasma excitation and weak spectral line emissions in LIBS, leading to diminished characteristic line intensities and heightened matrix effects. Consequently, standalone LIBS-based quantitative analysis of sulfur remains difficult. In contrast, IR spectroscopy excels in capturing the chemical states of sulfur within specific functional groups through its sensitivity to molecular vibrational modes. By synergizing LIBS’s atomic excitation properties with IR’s molecular structural resolution, dual-scale atomic-molecular characterization of nonmetallic elements is achieved in this study, enabling enhanced quantitative precision. The NIST database is referenced to identify spectral line features associated with sulfur in the LIBS spectrum. The relevant spectral lines are presented in Figure 9a. As depicted in Figure 9a, sulfur exhibits weak LIBS emission lines suitable for quantitative analysis but it demonstrates distinct absorption bands in IR spectra (see dashed boxes in Figure 9b, which indicate previously known sulfur spectral peaks). Therefore, multimodal data integration enables comprehensive material characterization by leveraging complementary analytical perspectives. It is important to note that sulfates are known to exhibit characteristic bands near 1250–1080 cm^−1^ (asymmetric O–S stretching vibration) and 680–580 cm^−1^ (asymmetric O–S–O bending vibration), which is consistent with the observation in Figure 9b. However, the characteristic bands around 3400 cm^−1^ and 1600 cm^−1^ in the figure are likely attributable to the vibrational characteristics of hydrates (specifically the O-H stretching vibration and O-H bending vibration), rather than those of sulfates or other sulfur species.

In multimodal learning, the fusion of heterogeneous spectral features facilitates deeper insights into intrinsic data relationships and cross-modal correlations, thereby significantly improving analytical accuracy. Moreover, the use of multimodal data for model training effectively enhances generalization performance [33]. Specifically, by learning and integrating multi-source data information, the model constructs more universal feature representations, thus achieving enhanced adaptability to unknown data distributions.

**Figure 10:**
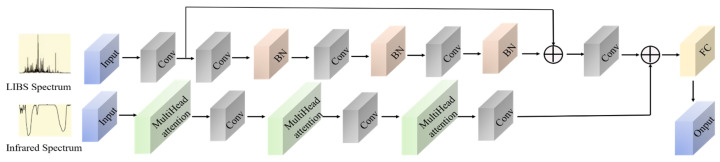
MCNN network architecture diagram.

This study develops a dual-input MCNN that integrates two neural architectures: a residual neural network for LIBS data processing and a multi-head self-attention mechanism for IR spectral analysis. The outputs are fused through fully connected layers to generate the final predictions. The proposed model synergistically integrates LIBS spectral features with IR spectral characteristics, thus enhancing analytical and predictive performance. The architectural schematic is presented in Figure 10.

Residual neural networks introduce “skip connections” or “residual connections,” which directly link the input to the output of a given layer, allowing the network to learn the residual between the input and output. This design ensures that information is transmitted directly to deeper layers [34]. This architectural innovation enables effective backpropagation in deep networks, mitigating gradient vanishing/explosion phenomena, and enhancing training efficiency and noise robustness. As illustrated in Figure 10, residual connections are implemented within the LIBS processing stream to suppress gradient oscillations induced by high-frequency spectral noise. Concurrently, the feature reuse mechanism enhances the ability to analyze weak spectral lines, strengthening the LIBS spectral learning capacity.

The multi-head self-attention mechanism utilizes parallel attention heads to independently compute weighted sums for distinct subsequences of the input, enabling multi-perspective comprehension and representation of input correlations, thus capturing subspace-specific information. This architecture enhances the model’s adaptability and robustness to data uncertainty and noise through diversified feature learning across attention heads. Furthermore, its parallelized computational framework significantly improves processing efficiency by handling multiple information streams concurrently. Infrared spectral data, as high-dimensional sequences, often contain key features distributed within local absorption peaks and global correlations between peak groups. The multi-head self-attention mechanism, through the parallelization of multiple independent attention heads, can simultaneously capture spectral features at different levels. This mechanism effectively suppresses baseline drift and noise interference via dynamic weight allocation. Furthermore, it enhances weak peak signals, offering a multi-scale representation foundation for precise quantitative analysis.

After feature extraction from both LIBS and infrared spectral data, a fully connected layer is used to fuse the output features from the two neural networks. This fully connected layer is responsible for learning how to optimally combine the features from LIBS and infrared spectra. Finally, the fused features are fed into the final regression layer to complete the regression task.

### 3.3. Feature Selection Methodology

High-dimensional spectral data acquired through LIBS are prone to the curse of dimensionality, which can exacerbate overfitting risks. Consequently, identifying wavelength subsets that are strongly correlated with sulfur constitutes a critical preprocessing step. Feature selection reduces data dimensionality while enhancing model efficiency and generalizability. Feature selection algorithms can be categorized into filter, wrapper, and embedded methods, with the selection of the algorithm tailored to dataset characteristics to optimize model performance [35].

This study employs three feature selection algorithms—Random Forest (RF, wrapper-type, embedded-type), Competitive Adaptive Reweighted Sampling (CARS, embedded-type), and Extreme Gradient Boosting (XGBoost)—to identify optimal spectral feature subsets through comparative experimental validation. The following sections provide details of these algorithms:

(1) The Competitive Adaptive Reweighted Sampling (CARS) method is a feature selection algorithm based on Monte Carlo sampling and partial least squares regression (PLSR). It utilizes the absolute values of regression coefficients as variable weights, iteratively selecting features with significant contributions to the target variable through an exponential decay function and adaptive reweighted sampling. This process reduces feature dimensionality and optimizes the model. CARS effectively handles high-dimensional, complex, and redundant spectral data thereby improving the accuracy and stability of regression predictions.

We let n represent the initial number of spectral features. During each sampling iteration, a subset of samples is randomly selected as the training set, with the remainder serving as the test set. A PLS model is then established, as shown in Equation (1):

(1) $$y_{i}=\overset{q}{\underset{j=1}{\sum }}t_{ij}w_{j}+\epsilon_{i}$$

In this context, $y_{i}$ represents the true value of the *i*th sample in the PLS model, $t_{ij}$ denotes the score of the *i*th sample on the *j*th principal component, $w_{j}$ is the weight of the *j*th principal component, and ϵ signifies the error term in the PLS model.

The exponential decay function f(i) eliminates wavelengths with low absolute regression coefficient weights (p), retaining only significant feature wavelengths. During each sampling iteration, Adaptive Reweighted Sampling (ARS) selects n×f(i) wavelength variables for PLS modeling, followed by the calculation of the root mean square error (RMSE).

(2) $$f(i)=\frac{1}{1+e^{\left(\mu \left(i-k\right)\right)}}$$

(3) $$p_{j}=\frac{\left|\beta_{j}\right|}{\sum_{k=1}^{p}\left|\beta_{k}\right|}$$

Here, f(i) represents the functional form of the Exponential Decay Function (EDF), with μ and k as its constant parameters, while $p_{j}$ denotes the absolute regression coefficient weight of the *j*th feature wavelength. The corresponding mathematical formulations are provided in Equations (2) and (3).

After completing N sampling iterations, the CARS algorithm generates N candidate feature wavelength subsets and their associated RMSECV (Root Mean Square Error of Cross-Validation) values. The wavelength subset corresponding to the minimum RMSECV is selected as the optimal feature subset.

(2) The Random Forest (RF) algorithm is an ensemble learning method based on decision trees. By constructing multiple decision trees, the random forest enhances prediction accuracy and enables feature selection through the evaluation of feature importance across individual trees. In RF-based feature selection, the importance of each feature is quantified by its frequency of usage across all constituent decision trees. This frequency metric can be expressed as shown in Equation (4):

(4) $$I_{count}\left(X_{j}\right)=\overset{T}{\underset{t=1}{\sum }}\overset{Nt}{\underset{n=1}{\sum }}I\left(X_{j}=K_{n,t}\right)$$

where $X_{j}$ denotes the jth feature, T represents the total number of trees in the random forest, $N_{t}$ indicates the node count in the tth tree, and $K_{n,t}$ specifies the feature utilized at the nth node of the tth tree. This metric facilitates the identification of highly predictive features, thereby enabling the selection of optimal wavelength subsets.

(3) Extreme Gradient Boosting (XGBoost) is an ensemble learning algorithm based on gradient boosting. During the construction of trees, XGBoost calculates feature importance scores to guide feature selection. In this study, the feature importance score is determined by the frequency with which a feature is used in node splitting across all trees, as formulated in Equation (5):

(5) $$Weight_{i}=\overset{n}{\underset{j=0}{\sum }}x_{i}=x_{ij}$$

where $Weight_{i}$ denotes the weight of feature i, $x_{i}$ represents feature i, $x_{ij}$ corresponds to the feature at the jth splitting node, n is the total number of tree nodes, and i is an indicator function that equals 1 when $x_{i}=x_{ij}$ and 0 otherwise.

Indeed, CNNs can implicitly perform feature selection through convolutional filters. One of the main reasons for explicitly selecting features prior to applying CNNs in this study is the difference between spectral data and image data. Unlike image data, spectral data typically come with clear prior knowledge, particularly in chemical analysis, where relevant features have known wavelength positions and intensity–concentration relationships. As this study focuses on the quantitative analysis of sulfur elements, we aim to incorporate this prior knowledge into the model to enhance its learning effectiveness. This approach improves the model’s performance and reduces the interference of irrelevant or redundant features. Explicit feature selection facilitates the removal of redundant features and noise, allowing the MCNN model to focus on the most discriminative features, thereby enhancing its stability and accuracy.

One key reason for performing feature selection solely on the LIBS spectra, rather than on the infrared spectra, is that the features in the infrared spectra directly related to sulfur content are highly localized and chemically significant. These key absorption peaks occupy narrow wavelength ranges, resulting in a naturally lower effective feature dimension. In contrast, the LIBS spectra contain thousands of emission lines, which exhibit significant redundancy and background noise. Given that infrared spectra contain fewer redundant features than LIBS spectra, feature selection is essential for extracting sulfur-related signals from the LIBS spectra. The use of full spectral data as input allows for the retention of more critical information from the infrared spectra, without negatively impacting the model’s performance. Additionally, the multi-head attention module, utilized in the infrared data branch, dynamically focuses on discriminative regions during the training process, thereby performing implicit feature selection.

## 4. Results

To evaluate the effectiveness of different feature selection algorithms on the multimodal regression model, this study analyzes regression results obtained using full LIBS spectra versus wavelength subsets selected by various feature selection methods. Furthermore, to validate the performance of the proposed multimodal approach for predicting sulfur content in Martian rocks, prediction results from three single-modal regression models (which utilize LIBS spectra exclusively) are incorporated for comparative assessment.

The regression model’s performance is evaluated using the Root Mean Square Error of Prediction (RMSEP) and the Coefficient of Determination (R^2^), as defined in Equations (6) and (7). In order to comprehensively assess the model’s stability, five-fold cross-validation was employed in this work.

(6) $$RMSEP=\sqrt{\frac{1}{n}\sum_{i=1}^{n}{\left(y_{i}-{\hat{y}}_{i}\right)}^{2}}$$

(7) $$R^{2}=\;1-\frac{\sum_{i=1}^{n}{\left(y_{i}-\hat{y_{i}}\right)}^{2}}{\sum_{i=1}^{n}{\left(y_{i}-\bar{y_{i}}\right)}^{2}}$$

Here, *n* denotes the sample size, $y_{i}$ represents the actual value, and ${\hat{y}}_{i}$ is the predicted value which signifies the mean value of the samples. A lower RMSEP and an R^2^ closer to one indicate higher predictive accuracy, reflecting stronger alignment between model predictions and experimental measurements.

### 4.1. Performance Comparison Between Single-Modal and Multimodal Models

**Figure 11:**
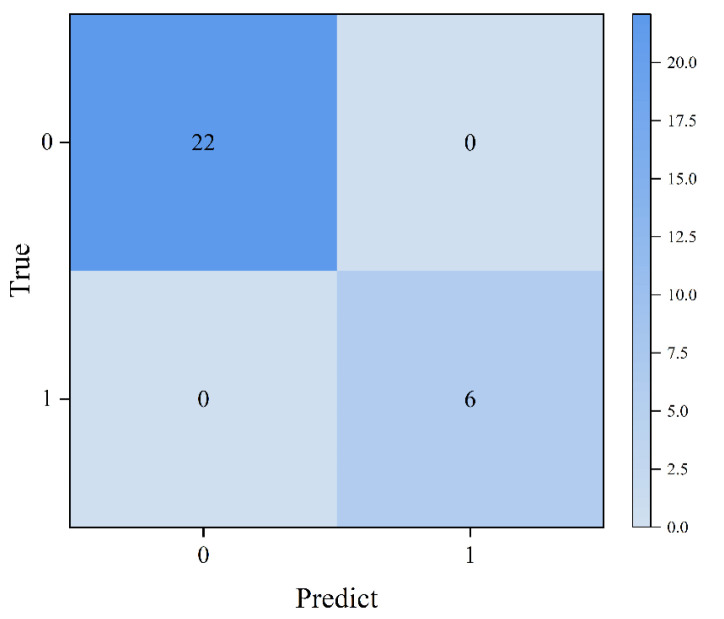
Confusion matrix of sample classification results.

For the multimodal model, IR spectra were first input into the classification network, achieving 100% classification accuracy, and the corresponding confusion matrix is shown in Figure 11. Based on the sulfur content distribution, samples with sulfur concentrations below 9.13 wt% were labeled as the low-content group, while those exceeding 9.13 wt% were designated as the high-content group.

The performance of common machine learning classification models, such as Support Vector Machine (SVM), RF, and K-Nearest Neighbor (KNN), in the infrared spectral classification task was further explored to validate the performance of the improved ViT model. The experimental results are presented in Table 3, where it can be observed that these models perform less effectively on our infrared spectral dataset compared to the proposed improved ViT model.

It is hypothesized that the enhanced performance of the modified ViT model stems from three key factors. First, the self-attention mechanism inherent in the ViT model effectively captures complex global dependencies among samples. This capability is particularly advantageous for high-dimensional spectral data, resulting in a significant improvement in classification accuracy. Second, infrared spectral data contain locally correlated absorption peaks that are critical for distinguishing sulfur content. The integration of convolutional operations into the encoder allows the model to synergistically combine the local inductive bias of CNNs with the long-range dependency modeling of ViT, thereby enabling precise localization of discriminative spectral regions. Finally, the ViT model demonstrates robust feature-learning capabilities, enabling the discernment of subtle differences in infrared spectral data and addressing the limitations of traditional methods (e.g., SVM, RF, KNN) in resolving fine-grained variations.

Following classification, LIBS and IR spectral data for the corresponding samples were fed into their respective regression models to generate experimental results. The parameters of the MCNN for both groups are provided in Table 4 and Table 5.

To validate the performance of the proposed method, comparative analyses were conducted with three single-modal approaches: Inception-v2 network, BPNN, and SVR. Notably, these methods directly utilized LIBS spectral data for sulfur content prediction without prior classification using NIR spectra, representing single-modal data strategies.

**Figure 12:**
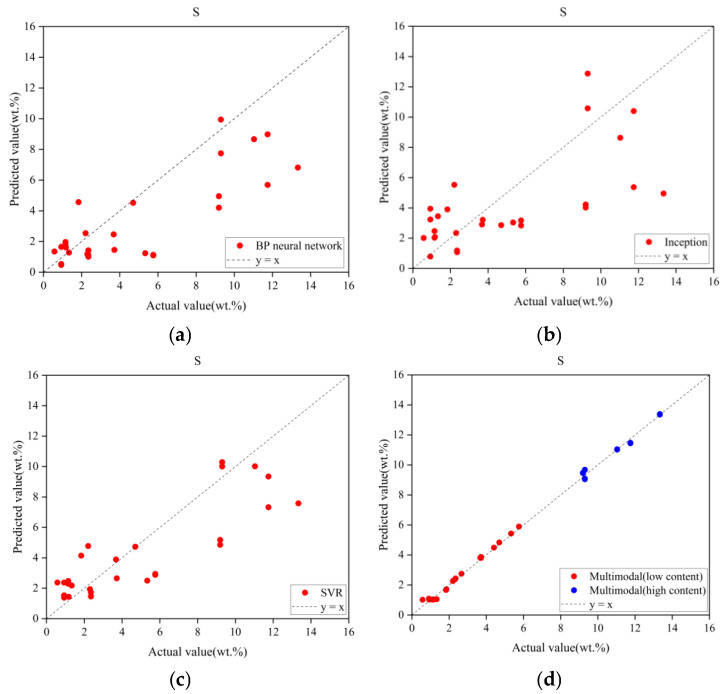
Prediction results of multimodal model and unimodal model. (**a**) BP network prediction results. (**b**) Inception network prediction results. (**c**) SVR network prediction results. (**d**) Multimodal model prediction results.

The experimental results of the proposed multimodal method and the three comparative approaches are summarized in Table 6 and Figure 12. Comparative analysis clearly demonstrates that the multimodal model achieved the smallest RMSEP and higher R^2^ values, indicating superior predictive performance compared to the single-modal methods.

In Figure 12, the horizontal axis represents the actual sulfur content of the samples, and the vertical axis corresponds to the predicted sulfur content. The dashed line represents the ideal reference line (1:1 ratio). Prediction accuracy increases as data points cluster closer to this line. It is important to note that the results presented in Figure 12 and Table 5 reflect the model’s performance on the validation subsets following five-fold cross-validation.

The BP neural network exhibited the lowest RMSEP among the three unimethods, indicating the smallest error in its predictions. This can be attributed to its ability to fit complex data through multi-layer non-linear mappings, thereby enhancing its predictive ability. However, it demonstrates a systematic underestimation in the high-concentration range. In comparison, the Inception network demonstrates greater dispersion, poorer linearity, and more pronounced outliers in low-concentration samples. The SVR model performs better in the 2–6 wt% range, exhibiting a superior linear relationship thanks to its non-linear high-dimensional regression that reduces overfitting and results in a higher R^2^. However, it continues to underperform in the higher concentration range, demonstrating significant systematic underestimation.

In Figure 12d, the red and blue points represent the predicted results of the MCNN model for the low-content and high-content groups, respectively. From the figure, it is evident that, regardless of concentration, the multimodal model demonstrates strong convergence, with most data points closely aligned along the ideal trajectory. This underscores the superior performance of the multimodal approach in quantitative analysis.

This is due to the MCNN network’s integration of both LIBS and infrared spectral information, extracting useful features from each data source, which enables the model to achieve a more comprehensive and accurate feature representation. By combining information from both sources, the model reduces the errors and limitations associated with using a single spectrum, such as noise and spectral interference.

The visual comparison reveals that single-modal model predictions exhibit significant dispersion, whereas the multimodal model shows strong convergence, with most data points aligning tightly along the ideal trajectory.

### 4.2. Experimental Results of MCNN Models with Different Feature Subsets

As demonstrated in Section 4.1, the multimodal model outperforms all other methods. While LIBS spectral data play a critical role in regression tasks, their high dimensionality (as discussed in Section 3.3) can exacerbate overfitting risks and compromise model performance. Appropriate feature selection methods mitigate the curse of dimensionality, and this section presents the regression performance under different feature selection algorithms.

For the RF model, the maximum tree depth (max_depth) was set to none to ensure node expansion until all leaf nodes contained fewer than two samples. Since the RF model outputs feature importance rankings rather than directly determining feature counts, the number of features was manually specified as 150 in this experiment.

For the CARS algorithm, parameters were configured to balance model simplicity and training efficiency: 50 Monte Carlo iterations, 20 maximum principal components, and 10 cross-validation folds. Feature subsets corresponding to the iteration with the minimal root mean square error were selected as optimal, and exponential decay was monitored throughout the process.

The XGBoost model, similar to the RF model, generates feature importance rankings. The optimal feature count was initially set to 200, with additional hyperparameters including a maximum tree depth of 6 and a learning rate of 0.3.

**Figure 13:**
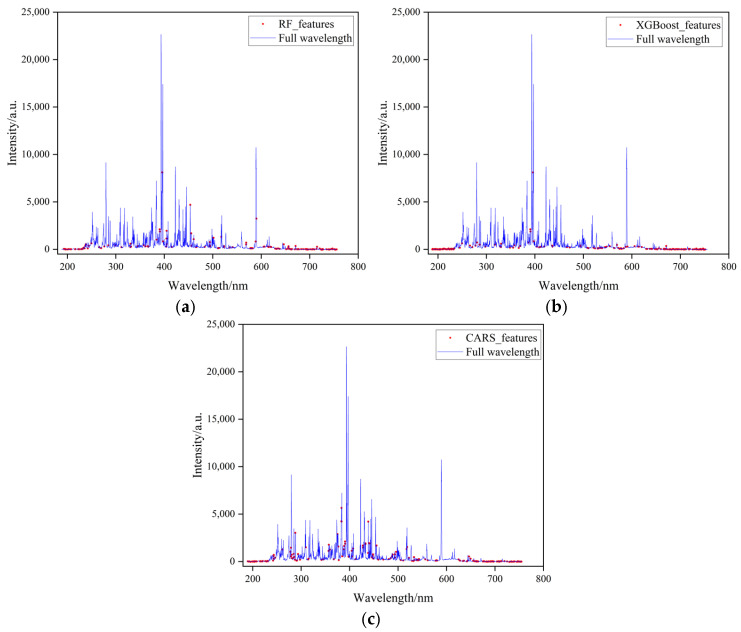
Feature subsets selected by different feature selection algorithms. (**a**) RF algorithm feature selection results. (**b**) XGBoost algorithm feature selection results. (**c**) CARS algorithm feature selection results.

Figure 13 illustrates the features selected by each algorithm, with red dots denoting selected features and blue lines representing full spectral data.

**Figure 14:**
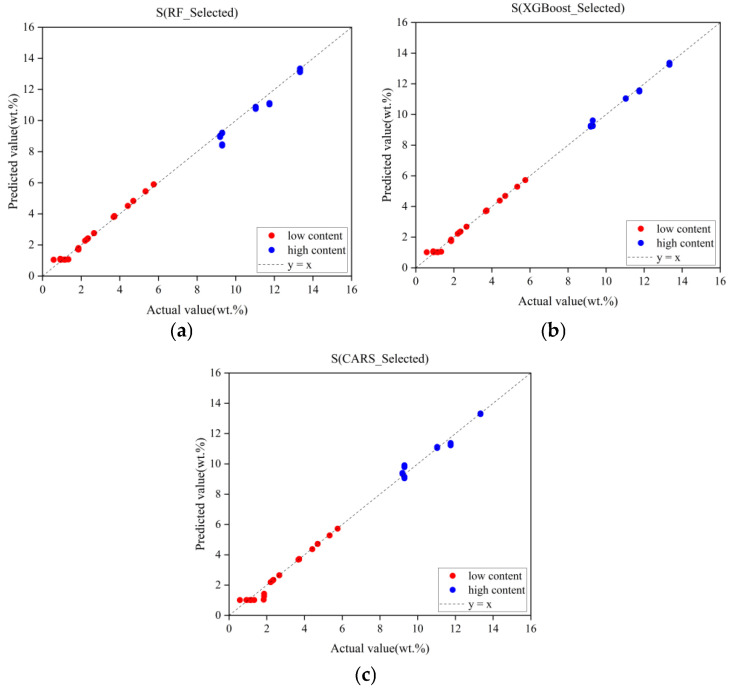
Prediction results of MCNN under different feature subsets. (**a**) RF feature subset prediction results. (**b**) XGBoost feature subset prediction results. (**c**) CARS feature subset prediction results.

The selected LIBS spectral features were input into the MCNN model, and experimental results from different feature selection algorithms are summarized in Table 7 and Figure 14. The results presented here also reflect the model’s performance on the validation subsets following five-fold cross-validation. The red and blue points represent the predicted results of the MCNN model for the low-concentration and high-concentration groups, respectively. As shown in the table, features selected by the RF algorithm improved regression performance for low-concentration samples but degraded performance for high-concentration samples. This can be attributed to the fact that in low-concentration samples, the sulfur element’s LIBS spectral line intensity is low, making it more susceptible to noise interference. The RF algorithm selects a stable subset of features through a voting mechanism across multiple decision trees, effectively suppressing related interferences. However, at higher concentrations, the increased sulfur spectral line intensity may be accompanied by self-absorption effects and baseline drift, which complicates the prediction process. The RF algorithm was unable to effectively capture the nonlinear relationships, leading to increased prediction deviations in the high-concentration range. Conversely, the CARS algorithm enhanced regression outcomes for high-concentration samples while compromising the model’s performance for low-concentration samples. This can be attributed to the decreased signal-to-noise ratio in low-concentration samples, which misled CARS due to random noise. In contrast, features selected by XGBoost improved regression accuracy for both concentration groups, demonstrating the most significant performance gains among all evaluated methods. This superiority arises from XGBoost’s gradient-boosted feature selection mechanism, which employs an adaptive weight allocation strategy to identify feature subsets with strong physicochemical relevance from high-dimensional LIBS data. Unlike redundant spectral wavelengths that introduce noise in full-spectrum analysis, XGBoost prioritizes feature regions closely associated with target element excitation transitions through dual evaluation criteria: information gain and coverage rate. Simultaneously, it effectively eliminates pseudo-correlated variables caused by baseline fluctuations, thus significantly enhancing model performance.

### 4.3. Ablation Study

To validate the efficacy of the proposed method, four additional control experiments were conducted using the original dataset. The first group bypassed the IR spectral classification step, directly inputting both LIBS and IR spectra into the MCNN model for performance analysis. The second group removed the IR spectral input channel, retaining only LIBS spectra for regression. The third group mirrored the second, but excluded LIBS spectra, relying solely on IR data.

Given that spectral fusion strategies can be categorized into data-level fusion (low-level), feature-level fusion (mid-level), and decision-level fusion (high-level), a fourth experiment was designed to compare performance by concatenating LIBS and IR spectra (end-to-end linkage) and inputting them into an Inception network, thus evaluating data-level fusion against the proposed decision-level fusion strategy. All experiments employed identical base architectures and training protocols, with modifications limited to core components.

**Figure 15:**
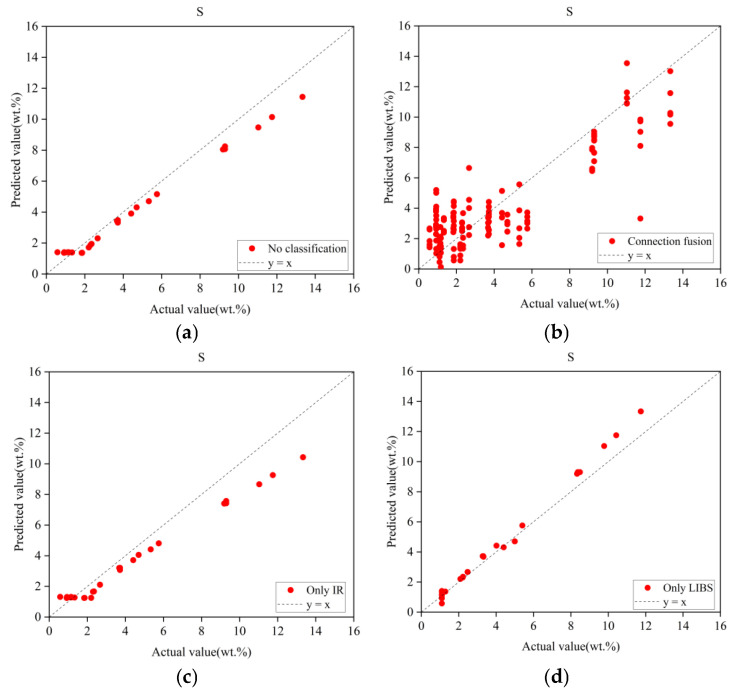
Prediction results under different ablation strategies. (**a**) MCNN prediction results without pre-classification. (**b**) Spectral cascade fusion prediction results. (**c**) MCNN prediction results with only IR spectral input. (**d**) MCNN prediction results with only LIBS spectral input.

The results, summarized in Table 8, clearly demonstrate the superiority of the complete model over all ablated variants. The concatenated data-level fusion strategy exhibited significantly poorer performance compared to the decision-level fusion approach. Furthermore, LIBS spectra were found to contribute most substantially to regression accuracy, which is consistent with earlier findings. For visual verification, predicted versus actual values are plotted in Figure 15, which reinforces these conclusions.

**Figure 16:**
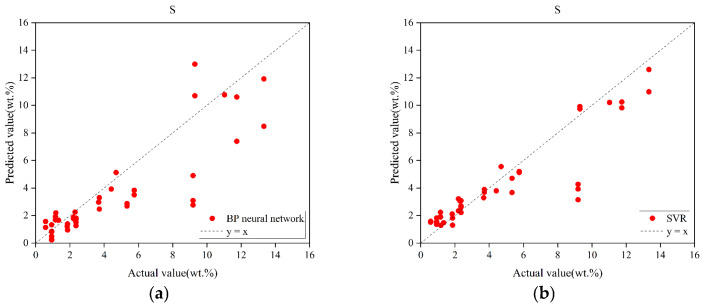
Prediction results of SVR and BPNN models under spectral concatenation fusion. (**a**) Prediction results of BPNN under spectral concatenation fusion. (**b**) Prediction results of SVR under spectral concatenation fusion.

It is noteworthy that in previous unimodal modeling, the performance of various base models, including BPNN, SVR, and Inception, was evaluated. However, the Inception model did not yield the highest predictive accuracy. To further investigate the impact of concatenated spectra on the model’s predictive capability, the concatenated spectra were input into both the BPNN and SVR models. The experimental results are shown in Table 9. For ease of analysis, the results of BPNN and SVR were also visualized, as shown in Figure 16. Based on the experimental findings, it is evident that although the SVR model outperforms the Inception model in terms of RMSEP and R^2^ metrics, its performance still lags behind that of the MCNN model, which utilizes a decision-level fusion strategy. This demonstrates that, within the context of this study, decision-level fusion outperforms data-level fusion. Furthermore, compared to the use of only the LIBS spectra, all three models exhibit a notable improvement in performance, accompanied by a reduction in the systematic underestimation in the high-concentration range. This further validates that the fusion of LIBS and infrared spectra allows the model to extract more information from the complementary feature space provided by multimodal data, thereby enhancing its predictive capacity.

The performance enhancement of the multimodal approach arises from the complementary physical information mechanisms of dual spectroscopy: the LIBS channel captures sulfur’s atomic emission characteristics through spectral line intensity ratios while the IR channel resolves sulfate speciation via antisymmetric stretching vibration peaks. The decision-level fusion network dynamically weights the contributions of both spectral modalities, effectively suppressing nonlinear response deviations caused by signal-to-noise ratio (SNR) degradation in single-modal analysis.

Notably, cation species introduce interference in sulfate identification due to their modulation effects on molecular vibrational modes, inducing spectral peak shifts. The proposed MCNN addresses this challenge by isolating cation-induced interference factors through cross-modal feature extraction, thus improving prediction robustness. This demonstrates that the multimodal fusion strategy not only enhances intrinsic sulfur signal extraction but also mitigates spectral interference from cation-induced peak shifts.

## 5. Conclusions

This study investigated the detection of sulfur content in rocks using multimodal spectral data with the aim of providing potential solutions for sulfate detection in Martian exploration missions. The methodology comprises three critical phases: initial classification of samples using IR spectral data, followed by the training of a purpose-built multimodal convolutional model (MCNN) integrating LIBS and IR spectral inputs, and concluding with quantitative prediction of sulfur content in rock specimens.

To validate the proposed method, its performance was systematically compared with three conventional LIBS regression models: BPNN, SVR, and Inception-v2. Experimental results demonstrate that the MCNN model outperforms all baseline methods in both RMSEP and R^2^. This superiority highlights the enhanced learning capacity of the multimodal approach, in which complementary spectral modalities synergistically improve model performance by leveraging atomic (LIBS) and molecular (IR) information.

Our experimental findings reveal that while CNNs possess strong feature extraction capabilities, the high dimensionality of LIBS spectral data—coupled with substantial noise and redundant features—may limit their full potential under the current experimental constraints. To further enhance the performance of the MCNN model, three distinct feature selection algorithms were implemented to screen the full LIBS spectra, identifying feature subsets strongly associated with sulfur content. These optimized subsets were then used for MCNN model training, replacing the full LIBS spectra. Comparative analysis demonstrated that the feature subset selected by the XGBoost algorithm delivered the most significant performance improvement for the MCNN model, whereas subsets chosen by the RF and CARS algorithms partially degraded model efficacy.

Our experimental findings demonstrate that regardless of whether LIBS data undergo feature selection, the prediction accuracy for samples with sulfur content between 0 and 1 wt% remains inferior compared to higher-concentration samples, despite overall satisfactory performance. We attribute this limitation to the extremely low sulfur content in these samples, which results in excessively weak characteristic spectral line intensities in their LIBS spectra. This insufficient signal intensity hampers the model’s capability to extract discriminative features, thus compromising the regression outcomes.

Subsequently, to further validate the effectiveness of different modules in our proposed methodology, four comparative experiments were conducted. The results demonstrated that our integrated approach consistently achieved superior performance overall.

Notably, characteristic spectral lines of certain anions were found to be nearly undetectable in practice. Based on our experimental findings, we proposed that anion detection could alternatively be accomplished by analyzing correlations between anions and other detectable spectral lines. This hypothesis aligns with the operational mechanism of CNNs, which effectively extract such latent feature correlations to perform anion analysis tasks.

## Figures and Tables

**Table 1:** Types of rock matrix and evaporite minerals.

Rock Matrix	Sulfate
Basalt GBW07105	CaSO_4_
	CaSO_4_·1/2H_2_O
	CaSO_4_·2H_2_O
	K_2_SO_4_
Andesite GBW07104	MgSO_4_
	FeSO_4_
	CaSO_4_·2H_2_O

**Table 2:** Information table of partial mass fraction (10^−2^ wt%) of rock matrix reference materials.

Component	Basalt GBW07105	Andesite GBW07104
SiO_2_	44.64 ± 0.11	60.62 ± 0.14
Al_2_O_3_	13.83 ± 0.13	16.17 ± 0.12
CaO	8.81 ± 0.09	5.20 ± 0.07
H_2_O^+^	2.86 ± 0.13	(1.5)
S	0.01	0.0192 ± 0.0021

Note: Values following “±“ represent measurement uncertainties, with reference values provided in parentheses.

**Table 3:** Classification Results for Common Machine Learning Algorithms.

Model	Accuracy
SVM	0.93
RF	0.90
KNN	0.83

**Table 4:** Parameter settings of the convolutional layers in the LIBS input stream.

Layer Name	Kernel Size	Channels	Dropout Rate
Conv1	1 × 3	4	
Conv2	1 × 3	64	
Conv3	1 × 5	128	0.2
Conv4	1 × 5	64	
Conv5	1 × 3	64	

**Table 5:** Parameter settings of convolutional layers and attention mechanism in the IR input stream.

Layer Name	Kernel Size	Num_Heads	Channels
Multi-Head Attention		4	
Conv1	1 × 3		8
Multi-Head Attention		4	
Conv2	1 × 5		32
Multi-Head Attention		4	
Conv3	1 × 3		64

**Table 6:** Performance of the multimodal model compared to the unimodal model.

Modal	RMSEP	R^2^
SVR	2.28	0.670
Inception-v2	2.91	0.466
BPNN	1.44	0.506
Multimodal (low content)	0.04	0.981
Multimodal (high content)	0.11	0.932

**Table 7:** MCNN model results under different feature subsets.

	Low Content			High Content		
	RF	CARS	XGBoost	RF	CARS	XGBoost
RMSEP	0.02	0.0687	0.02	0.15	0.093	0.06
R^2^	0.986	0.964	0.989	0.899	0.936	0.962

**Table 8:** Ablation experiment results.

	RMSEP	R^2^
No classification	0.61	0.956
Connection fusion	3.42	0.748
Only LIBS	0.34	0.964
Only IR	1.34	0.900

**Table 9:** Results of concatenated spectra under different models.

Model	RMSEP	R2
BPNN	2.73	0.691
Inception-v2	3.42	0.748
SVR	1.29	0.827

## Data Availability

Data are contained within the article.

## Supplementary Materials

The following supporting information can be downloaded at: https://www.mdpi.com/article/10.3390/s25144388/s1, Table S1. The Certified Values and Uncertainties of Rock Component Analysis Standard Reference Materials.
