# Supplementary material for: Quantitative Analysis of Sulfur Elements in Mars-like Rocks Based on Multimodal Data
Source: Sensors (Basel). 2025 Jul 14;25(14):4388. doi: 10.3390/s25144388 (PMC12298777; doi:10.3390/s25144388)
Supplement: Supplementary file 1 [file sensors-25-04388-s001.zip › sensors-3648637-supplementary.pdf]

sensors MDPI

Supplementary materials

Table S1. The Certified Values and Uncertainties of Rock Component Analysis Standard Reference Materials

**Mass fraction (10^{-6})**	**Andesite GBW07104**	**Basalt GBW07105**
Ag	0.071±0.009	0.040±0.008
As	2.1±0.4	(0.7)
Au	(0.00095)	(0.00066)
B	4.7±0.8	3.5±1.0
Ba	1020±45	527±26
Be	1.1±0.2	2.5±0.4
Bi	0.081±0.016	0.048±0.017
Cd	0.061±0.014	0.067±0.016
Ce	40±3	105±8
Cl	(46)	(114)
Co	13.2±1.0	46.5±3.4
Cr	32±3	134±11
Cs	2.3±0.7	(0.7)
Cu	55±3	49±3
Dy	1.85±0.17	5.6±0.3
Er	0.85±0.13	2.0±0.2
Eu	1.02±0.05	3.2±0.2
F	280±25	700±44
Ga	18.1±1.4	24.8±0.9
Gd	2.7±0.4	8.5±0.6
Ge	0.93±0.15	0.98±0.21
Hf	2.9±0.5	6.5±0.8
Hg	0.012±0.003	0.006±0.002
Ho	0.34±0.03	0.88±0.04

Table S1. The Certified Values and Uncertainties of Rock Component Analysis Standard Reference Materials (Continued)

Mass fraction (10^{-6})	Andesite GBW07104	Basalt GBW07105
I	(0.14)	
In	0.037±0.013	0.064±0.022
La	22±2	56±5
Li	18.3±0.9	9.5±0.9
Lu	0.12±0.03	0.19±0.05
Mn	604±18	1310±61
Mo	0.54±0.09	2.6±0.2
Nb	6.8±1.4	68±8
Nd	19±2	54±4
Ni	17±2	140±7
P	1030±24	4130±122
Pb	11.3±1.8	(7)
Pr	4.9±0.4	13.2±1.3
Rb	38±3	37±4
S	192±21	(100)
Sb	0.12±0.04	(0.08)
Sc	9.5±0.7	15.2±1.2
Se	(0.04)	0.073±0.024
Sm	3.4±0.2	10.2±0.5
Sn	0.79±0.17	2.0±0.4
Sr	790±35	1100±64
Ta	0.40±0.10	4.3±0.6
Tb	0.41±0.05	1.2±0.2
Te	0.017±0.005	(0.022)
Th	2.6±0.3	6.0±0.8
Ti	3090±90	14200±400
Tl	0.16±0.05	(0.12)

Table S1. The Certified Values and Uncertainties of Rock Component Analysis Standard Reference Materials (Continued)

**Mass fraction (10^{-6})**	**Andesite GBW07104**	**Basalt GBW07105**
Tm	0.15±0.05	0.28±0.04
U	0.90±0.19	1.4±0.3
V	94±4	167±11
W	(0.45)	(0.4)
Y	9.3±1.2	22±4
Yb	0.89±0.13	1.5±0.4
Zn	71±5	150±10
Zr	99±11	277±20
**(10^{-2})**		
SiO_2	60.62±0.14	44.64±0.11
Al_2O_3	16.17±0.12	13.83±0.13
TFe_2O_3	4.90±0.06	13.40±0.19
FeO	2.39±0.07	7.60±0.13
MgO	1.72±0.06	7.77±0.17
CaO	5.20±0.07	8.81±0.09
Na_2O	3.86±0.07	3.38±0.05
K_2O	1.89±0.05	2.32±0.06
H_2O+	(1.5)	2.86±0.13
CO_2	3.47±0.07	(0.19)
LOI	4.44±0.12	(2.24)

Note: Values following "±" represent measurement uncertainties, with reference values provided in parentheses.
